# Association between Interleukin-10-1082 G/A and Tumor Necrosis Factor-**α** 308 G/A Gene Polymorphisms and Respiratory Distress Syndrome in Iranian Preterm Infants

**DOI:** 10.1155/2017/6386453

**Published:** 2017-02-16

**Authors:** Abolfazl Khoshdel, Soleiman Kheiri, Peyman Omidvari, Fahimeh Moradi, Majid Hamidi, Hossein Teimori

**Affiliations:** ^1^Clinical Biochemistry Research Center, Shahrekord University of Medical Sciences, Shahrekord, Iran; ^2^Department of Pediatrics, Shahrekord University of Medical Sciences, Shahrekord, Iran; ^3^Cellular and Molecular Research Center, Faculty of Medicine, Shahrekord University of Medical Sciences, Shahrekord, Iran; ^4^Subspecialty of Pediatrics, Department of Pediatrics, Shahrekord University of Medical Sciences, Shahrekord, Iran; ^5^Department of Medical Genetics, Shahrekord University of Medical Sciences, Shahrekord, Iran

## Abstract

Cytokine polymorphisms may contribute to the prevalence of respiratory distress syndrome. The present study was done to investigate the frequency of interleukin- (IL-) 10 and tumor necrosis factor- (TNF-) *α* gene polymorphisms and their association with the risk of RDS in preterm infants. One-hundred and nineteen patients with RDS and 119 healthy preterm infants were enrolled. PCR restriction fragment length polymorphism was used to determine the frequency of IL-10 and TNF-*α* genotypes at -1082 A and -308 A, respectively. One-hundred and nineteen out of 238 infants had RDS (50%). The age of the mothers and gestational age ranged 17–45 (mean: 28.6 ± 5.3) years and 24–34 (mean: 34.3 ± 2.38) weeks, respectively. Totally, 23 deaths were recorded in the RDS group. Incidence of TNF-*α*-308 A/A and TNF-*α*-308 G/A was 84% and 16%, respectively. TNF-a-308 G/G was not found in both groups. Prevalence of IL-10-1082 G/G and IL-10-1082 G/A variants was 65.5% and 34.5%, respectively. IL-10-1082 A/A was not found in both groups. The incidence of the allele G in the IL-10-1082 polymorphism was lower in RDS group (*P* < 0.05). We found that the risk of RDS was correlated to sex, gestational age, and IL-10-1082.

## 1. Introduction

Respiratory distress syndrome (RDS) occurs mainly in preterm infants and its incidence is inversely correlated with gestational age and birth weight. It is also known as hyaline membrane disease which is a breathing disorder of mainly premature babies. In healthy infants, the alveoli—the small, air-exchanging sacs of the lungs—are coated by surfactant, which is a soap-like material produced in the lungs as the fetus matures in preparation for birth. If premature newborns have not yet produced enough surfactant, they are unable to open their lungs fully to breathe which cause RDS [1, 2]. RDS is a multifactorial disease [2]. The risk of RDS rises with increasing prematurity. Babies born before 29 weeks of gestation have a 60 percent chance of developing RDS [1, 2], but babies born at full term rarely develop this condition. Maternal risk factors for preterm birth include previous preterm birth, periodontal disease, low maternal body mass, poor prenatal care, poverty, being uninsured, and being a member of a minority group [1, 2]. The risk of RDS increases with maternal diabetes, multiple births, cesarean delivery, asphyxia, cold stress, and maternal history of affected infant [1, 2].

The incidence of RDS in preterm male or white infants is higher than other groups of patients. RDS affects about 1 percent of newborn infants and is the leading cause of death in babies who are born prematurely [3, 4]. About 10 percent of premature babies in the United States develop RDS each year [3, 4].

Surfactant deficiency is the primary cause of RDS. Variation in the genetic profile of RDS can also influence the incidence of disease in infant populations [5–8]. The most talented candidates for the surfactant deficiency that have been identified to date lie in the genes coding for the lung-specific proteins and surfactant-associated genes and especially those for SP-A, SP-B, SP-C and SP-D proteins. Indeed, polymorphisms in these genes have been associated with susceptibility to RDS [9]. These 4 major proteins play an important role in the function of surfactant. Documented data revealed that occurrence of polymorphism in these genes is responsible for incidence of RDS [10]. Previous evidences showed that respiratory and intestinal inflammations were involved in occurrence of RDS [11, 12]. Decisive role of cytokines as important regulators of surfactant should also be considered [13]. Cytokines may be the regulators of surfactant metabolism in the preterm infant [10–13], with SP-A, SP-B, SP-C, and SP-D proteins being involved in the maintenance of an infection-free and inflammation-free lung [10–13]. Even low levels of proinflammatory cytokines in the pregnancy period can reduce the risk of RDS in premature infants [14]. Proinflammatory reactions are characterized by production of cytokines and inflammatory cells that increases permeability of alveolar capillaries which can be associated with disorder in lung function [15]. Postnatal activation of circulating neutrophils and lymphocytes is another indicator of systemic inflammatory reaction which may contribute to tissue injury in preterm infants with RDS [16]. Moreover, it has been shown that, in preterm infants with RDS, the activation of circulating polymorphonuclear leukocytes has a role in the pathogenesis of this syndrome [16, 17].

Interleukin- (IL-) 10 is an important immunoregulatory cytokine which controls inflammatory process by suppressing the expression of proinflammatory cytokines [18]. Documented data showed that preterm infants with RDS harbored the higher levels of IL-10 than those without RDS and full-term newborn [19]. Besides, infants who suffered from RDS had the higher levels of tumor necrosis factor- (TNF-) *α* than those without RDS [20]. Previous study demonstrated that the TNF-*α* was a potent inhibitor of surfactant protein-A [21]. In addition, IL-10 is an important anti-inflammatory cytokine that modulates proinflammatory cytokine such as TNF-*α* [22]. Therefore, anti- and proinflammatory cytokines are important factors in development of RDS in infants [23]. Promoter regions of some of the key cytokine genes contain polymorphisms which can directly influence production of cytokines [24]. Therefore, abnormal cytokine production derived from specific polymorphisms can have effect on development of RDS. The present study was aimed at investigating the distribution of IL-10 and TNF-*α* gene polymorphisms and their association with the risk of RDS in preterm infants.

## 2. Materials and Methods

### 2.1. Ethical Issues

The study was approved by the Ethical Committee of Research of the Hajar Hospital, Iran, and Shahrekord University of Medical Sciences (ethical code: 90-10-31) and informed consents were obtained from the families of the patients. All families gave written informed consents to participate in the study and answered a questionnaire about symptoms and history of their diseases. The authors tried to protect the life, health, dignity, integrity, rights to self-determination, privacy, and confidentiality of personal information of studied patients. Information regarding the demographic data, such as mother age, gestational age, sex, Apgar score, and the need for mechanical ventilator or surfactant, was recorded.

### 2.2. Samples

This cross-sectional, case-control study was done on Cellular and Molecular Research Center of Shahrekord University of Medical Sciences, Shahrekord, Iran. From September 2011 to May 2013, a total of two-hundred and thirty-eight cord blood samples were collected from preterm neonates born pediatrics hospitalized in Hajar Hospital, Iran. Premature infants were classified into two groups: preterm newborns without RDS (*n* = 119) and those with RDS (*n* = 119). The gestational age in both groups was less than 37 weeks.

### 2.3. Inclusion and Exclusion Criteria

The inclusion criteria for control group were healthy newborn with gestational age less than 37 weeks. The case group included preterm neonates with RDS. RDS in preterm newborns was diagnosed based on the clinical criteria including grunting, intercostal retraction, nasal flaring, cyanosis, and tachypnea and also radiological findings such as chest radiographs with a diffuse reticulogranular pattern and air bronchograms. Preterm newborns with thoracic and cardiac defects, continuous medication infusion, and genetic syndromes were excluded from the study.

### 2.4. Laboratory Tests

Cord blood samples were collected in tubes containing EDTA and were stored at −20°C until DNA extraction. Genomic DNA was extracted by standard phenol chloroform method [25]. DNA segments of TNF-*α* and IL-10 genes were amplified using primers IL-10F CCAGGTAGAGCAACACTCCT/IL-10R CTCTTACCTATCCCTACTTCCGC (156 bp) for IL-10 and TNF-*α* F AATAGGTTTTGAGGGCCATG/TNF-RTCATCTGGAGGAAGCGGTAG (234 bp) for TNF-*α*. The PCR mixture for IL-10 consisted of 0.2 *μ*M of each primer, 0.2 mM dNTPs, 1x PCR buffer, 2 mM MgCl2, and 0.2 *μ*L Taq polymerase (Sinagen, Iran) and for TNF-*α*, 0.2 *μ*M of each primer, 0.12 mM dNTPs, 1x PCR buffer, 2 mM MgCl2, and 0.2 *μ*L Taq polymerase in total volume of 25 *μ*L. IL10 was amplified under a thermal condition consisting of 95°C for 6 min followed by 37 cycles of 95°C for 30 s, 55.2°C for 30 s, and 72°C for 40 s and a final extension for 2 min at 72°C. For TNF-*α*, the thermal condition consisted of 95°C for 2 min followed by 34 cycles of 95°C for 30 s, 55°C for 30 s, and 72°C for 40 s and the final extension at 72°C for 2 min. The amplified products were separated by polyacrylamide gel electrophoresis followed by silver staining at 45 mA for 1.5 hours.

### 2.5. Genotyping of the Polymorphisms

Genotyping status of PCR products was determined by restrictive fragmented length polymorphism (RFLP) assay. PCR products of IL-10 and TNF-*α* were subjected to digestion with EarI and NcoI restriction enzymes (Fermentase, Germany), respectively. All tests were done according to the manufacturer's instruction. The products of digestion were analyzed on 8% polyacrylamide gel. EarI restriction enzyme cleaved the variant of IL-10-1082 G into two distinct fragments of 126 and 30 bp, while the wild type allele (156 bp) remained intact. The PCR product of TNF-*α* G-308 A variant was also digested by NcoI restriction enzyme and showed two distinct bands of 218 bp and 16 bp and wild type allele remained uncleaved (234 bp).

### 2.6. Statistical Analysis

Continuous variables are presented as mean ± SD and categorical ones as frequency. Comparisons between the two groups were made using the Chi-square and Fisher's exact tests for categorical variables and independent *t*-test for continuous variables. Logistic regression was performed to modify the association of IL-10-1082 genotype and sex and gestational age of pediatrics. Statistical analysis was done by SPSS version 18 software and *P* value of 0.05 was considered as statistically significant.

## 3. Results 

### 3.1. Study Population and Prevalence of RDS

Two-hundred thirty-eight infants participated in this study, of whom 119 had RDS (50%), 119 were controls, and 111 (46.6%) were male. The age of the mothers ranged 17–45 (mean: 28.6 ± 5.3) years. The gestational age ranged 24–34 (mean: 34.3 ± 2.38) weeks. The characters of the participants are shown in Table 1. We found that the mean distribution of the age of the mothers, gestational age, and frequency of boy patient in control and RDS groups were 29.2 ± 5.8 and 28.1 ± 4.7 years, 35.6 ± 0.71 and 33 ± 2.7 weeks, and 37.8% and 55.5%, respectively. Prevalence of normal vaginal delivery in control and RDS groups was 23.5% and 16%, respectively (*P* = 0.143). We also found that the mean gestational age was significantly lower in RDS patients than control group (*P* < 0.05). Infants with RDS had significantly lower Apgar score (*P* < 0.05). Need for mechanical ventilator and surfactant was not applicable to the control group. There were 23 deaths in the RDS group but no death was reported in the control group (*P* < 0.05).

### 3.2. Distribution of TNF-*α*-308 Polymorphism

**Figure 1:**
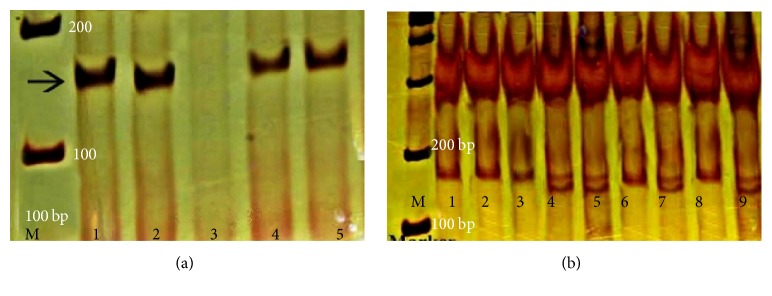
(a) PCR product of IL gene corresponding to a 156 bp band; M: 100 bp DNA ladder, lane 3: negative control, lanes 1, 2, 4, and 5: samples. (b) Genotyping of IL polymorphism by PCR-RFLP, M: 100 bp DNA ladder; subjects 1, 2, 3, 6, and 8: homozygotes for the G allele; subjects 4, 5, 7, and 9: heterozygotes for the A/G allele.

**Figure 2:**
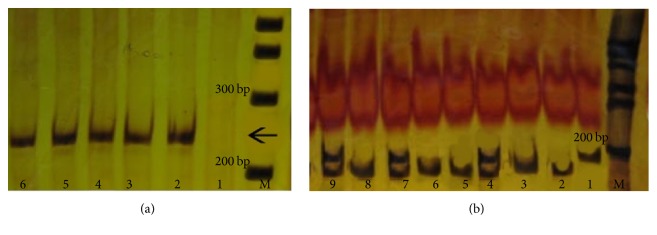
(a) PCR product of TNF gene corresponding to a 234 bp band; M: 100 bp DNA ladder, lane 1: negative control, lanes 2, 3, 4, 5, and 6: samples. (b) Digestion of of PCR product by NcoI, M: 100 bp DNA ladder; lane 1: homozygote for the G allele; lanes 4, 7, and 9: heterozygotes for the A/G allele; lanes 2, 3, 5, 6, and 8: homozygotes for the A allele.

Figure 1 represents the PCR product of IL gene and also genotyping of IL polymorphism by PCR-RFLP.  Figure 2 represents the PCR product of TNF gene and also digestion of PCR product by NcoI. The distribution of TNF-*α*-308 polymorphism in various groups of study is shown in Table 2. Total incidence of TNF-*α*-308 A/A and TNF-*α*-308 G/A was 84% and 16%, respectively. TNF-a-308 A/A and TNF-a-308 G/A prevalence were evaluated according to need of surfactant (92.9% and 7.1%), need of mechanical ventilation (93.9% and 6.1%), and death (95.7% and 4.3%). These three groups showed higher prevalence of TNF-a-308 A/A (*P* < 0.05) and lower prevalence of TNF-a-308 G/A (*P* < 0.05). TNF-*α*-308 G/G was not found in both groups.

### 3.3. Distribution of Interleukins Polymorphism

Distribution of IL-10-1082 polymorphisms in various studied groups is shown in Table 3. IL-10-1082 G/G and G/A prevalence were evaluated according to need of surfactant (57.1% and 42.9%), need of mechanical ventilation (57.6% and 42.4%), and death (56.5% and 43.5%). These three groups showed higher prevalence of IL-10-1082 G/A (*P* < 0.05) and lower prevalence of IL-10-1082 G/G (*P* < 0.05). Total prevalence of IL-10-1082 G/G and IL-10-1082 G/A variants was 65.5% and 34.5%, respectively. IL-10-1082 A/A was not found in both groups. IL-10-1082 G/A had a significantly higher incidence in the pediatric patients of RDS group (*P* < 0.05), while IL-10-1082 G/G had a significantly lower incidence in this group (*P* < 0.05).

### 3.4. Allele's Absolute Frequencies of TNF-*α*-308 and IL-10-1082 Polymorphisms

The allele's absolute frequencies of TNF-*α*-308 and IL-10-1082 polymorphisms in various studied groups are shown in Table 4. We found that the incidence of the allele G in the IL-10-1082 polymorphism was significantly lower in RDS group (*P* < 0.05). On the other hand, pediatric patients of the RDS group had the higher prevalence of TNF-*α*-308 A/A (*P* = 0.479) and IL-10-1082 G/A (*P* = 0.014).

### 3.5. Results Obtained from Logistic Regression

The result of logistic regression is shown in Table 5. We found that the risk of RDS was significantly related to the three variables of sex, gestational age, and IL-10-1082.

## 4. Discussion 

The present investigation was done in order to determine of the association of IL-10-1082 G/A and TNF-*α*-308 A promoter polymorphisms and incidence of RDS in a population of preterm infants in Iran. Our findings showed that the risk of RDS was significantly lower in preterm infants with the IL-10-1082 G/G genotype, suggesting a protective role of this variant in occurrence of RDS. We also found no significant association between TNF-*α* G-308A polymorphism and incidence of RDS in preterm newborns.

IL-10 is a significant immunoregulatory cytokine that is mostly produced by monocytes, macrophages, B cells, and T cells. It controls the inflammatory procedures by suppressing the expression of proinflammatory cytokines, chemokines, adhesion molecules, and antigen-presenting and costimulatory molecules in monocytes/macrophages, neutrophils, and T cells [18, 26–28]. Several investigations of complex diseases have indicated a main role for IL-10 in chronic inflammatory disorders that are considered by the predominance of cytokines such as IL-1, IL-6, IL-8, IL-10, IL-12, and TNF. These included Crohn's disease, psoriasis, multiple sclerosis, and rheumatoid arthritis [18, 26–28]. The exact mechanisms involved in the regulation of IL-10 production endure to be determined, although inherited factors seem to have imperative roles. A difference in IL-10 secretion in association with a Single Nucleotide Polymorphism (SNP) in the -1082 position of the gene promoter has been established. In particular, an association of the -1082 G allele with a high IL-10 producing ability has been shown, through assessments of both mRNA and protein [18, 26–28]. The IL-10-1082 G/A SNP is located within an Ets binding site. The -1082 A allele confers a higher binding empathy to the transcription factor PU.1, which inhibits gene expression and leads to decreased IL-10 expression in individuals carrying this allele [18, 26–28]; thus, it is reasonable that the IL-10-1082 GG and GA genotypes also have high expression rates in the premature lung.

G/G polymorphism of the IL-10-1082 has been reported to be a protecting factor against pulmonary diseases and especially acute RDS and active tuberculosis [29, 30]. Results of the previous investigations showed that IL-10-1082 G/G genotype had the lower incidence among critically ill patients with organ failure compared with healthy one [29, 30]. Significant associations between the presence of IL-10-1082 G/G polymorphism and lower incidence and mortality rate of diseases were found in patients who suffered from acute respiratory distress syndrome (ARDS) [29] which was similar to our findings.

G to A SNP at a position of 1082 are important for regulation of IL-10 transcription. Individuals with GG genotype have higher levels of IL-10 transcription. Besides, those with higher levels of GG genotype have also higher concentrations of circulating IL-10 in both in vivo and in vitro conditions [31, 32]. Lower levels of IL-10 were found in patients with acute RDS compared with those without ARDS [33] which was similar to our findings. Yanamandra et al. (2005) [34] reported that the IL-10-1082 A allele (for lower IL-10 production) had a minor effect on the combined outcome of death or bronchopulmonary dysplasia.

Our finding is similar to the result of Capasso et al. (2007) [23] which was conducted on Italian preterm newborn population. They showed that the risk of RDS was significantly lower in IL-10-1082 GG/GA positive preterm newborn pediatrics than those which were positive for A/A genotype. Results of the previous research reported the substantial anti-inflammatory effects of recombinant IL-10 in the cases of RDS [35] which was similar with our results. The balance between anti- and proinflammatory cytokines is important in response to injuries and acute RDS [36]. Imbalance between anti- and proinflammatory cytokines is responsible for increase in cytokine response and organ sequelae during neonatal infection in preterm infant [37].

Our results represented that infant patients of the RDS group had a significantly higher prevalence of TNF-*α*-308 A/A and IL-10-1082 G/A. Release of IL-10 (anti-inflammatory cytokines) has been shown to be upregulated by circulating TNF-*α* (proinflammatory cytokine) [37, 38]. IL-10 has also the ability to suppress the synthesis of proinflammatory cytokines and of effectively downregulating proinflammatory response [39]. IL-10 attenuates the proinflammatory response in sepsis and decreases mortality in some animal model [40]. Massive proinflammatory reaction with severe organ dysfunction will occur in the cases of anti- and proinflammatory cytokines imbalance [41].

Concentration of IL-10 in the bronchoalveolar lavage fluid of preterm infant ventilated due to RDS has been significantly raised over the first five postnatal days [42]. Exogenous administration of IL-10 will protect against excessive proinflammatory cytokine production and mortality [43]. Neutralization of endogenous IL-10 with monoclonal antibodies will increase the levels of proinflammatory cytokine and fatality rate [44].

We found that presence of IL-10-1082 G/A increased the susceptibility to RDS. IL-10-1082 G/A genotype is associated with lower production of IL-10 and also consequent increase in the proinflammatory cytokine is associated with occurrence of RDS. We also found that the incidence of IL-10-1082 G allele in RDs group was significantly lower than control. G allele has been associated with increase in IL-10 production and decrease in proinflammatory cytokine production which can reduce the risk of RDS [31].

This study also examined the effect of the TNF-*α*-308 A polymorphism on the risk of RDS. We found no significant difference in the incidence of genotype or allele between the control and RDS groups of preterm infants. However, the dramatic inhibitory effect of TNF-*α* on surfactant protein expression was also reported previously [21]; we found no significant association between the presence of TNF-*α*-308 A, IL-10-1082 G polymorphisms with wild type allele with need for mechanical ventilator and surfactant and mortality rate in preterm infants who suffered from RDS. Andalas et al. (2016) [45] reported that the concentration of TNF-*α* between preterm and control groups was not statistically different, 5.5 ± 2.9 mg/dL versus 10.1 ± 17.9 mg/dL, *P* = 0.112. They also showed that the level of TNF-*α* had no strong association with either genotype distribution or allele frequency of SNP -308 G/A TNF-*α* which was similar to our findings. Gong et al. (2005) [46] represented that the TNF-*α*-308A and TNFB1 alleles were in linkage disequilibrium in the cases of acute RDS. They showed that these polymorphisms were not associated with acute RDS susceptibility which was similar to our results. The TNF-*α*-308A allele was associated with increased 60-day mortality in acute RDS, with the strongest association found among younger patients. They also reported that there was no association between the TNFB polymorphism and ARDS mortality. Li et al. (2013) [47] in their meta-analysis study reported that throughout twelve case-control studies and one cohort study analyzed, no association between TNF-*α*-308A/G polymorphism and pneumonia risk was observed for AA + AG genotypes. They also indicated that TNF-*α*-308A/G polymorphism was not associated with pneumonia mortality (*P*  =  0.07). Furthermore, there was no association of TNF-*α*-238A/G polymorphism with the risk of pneumonia (*P*  =  0.20). Similar results were reported by Kazzi et al. (2004) [48]. They showed that the genotypic distributions of lymphotoxin-*α*250 and TNF-*α*308 were comparable among the groups of infants. However, the AA and GA TNF-*α*238 genotypes were much less likely to occur among infants with bronchopulmonary dysplasia than among healthy infants. The adenine allele of TNF-*α*238 was absent among infants with severe bronchopulmonary dysplasia and occurred significantly less often among infants with moderate or severe bronchopulmonary dysplasia, compared with infants with mild bronchopulmonary dysplasia. They finally concluded that the adenine allele of TNF-*α*238 may reduce the risk and severity of bronchopulmonary dysplasia.

## 5. Conclusion

In conclusion, we found that some genotypes are associated with the risk of RDS. The IL-10-1082 G/G genotype significantly reduced the possibility of RDS and IL-10-1082 G/A genotype increased susceptibility to RDS, while no association was found between TNF-*α*-308 A polymorphism and RDS in preterm newborns. Our investigation also showed no association between the presence of TNF-*α*-308 A, IL-10-1082 G polymorphisms and wild type allele with need for mechanical ventilator and surfactant and mortality rate in preterm infants who suffered from RDS.

## Figures and Tables

**Table 1:** Some characters of infants in the study groups.

Variable	Control	RDS^*∗*^	*P* value
	(Mean ± SD^*∗∗*^)	(Mean ± SD)	
Mother age	29.2 ± 5.8	28.1 ± 4.7	0.109
Gestational age	35.6 ± 0.71	33 ± 2.7	<0.001
Sex (Boy)	45 (37.8)	66 (55.5)	0.006
Normal vaginal delivery	28 (23.5)	19 (16)	0.143
Gravid	2.27 ± 1.36	2 ± 1.15	0.139
Apgar score	8.8 ± 0.5	7.5 ± 1.6	<0.001
Duration (day)	8.3 ± 1.9	11.5 ± 7.7	0.312
Surfactant	0	28 (23.5)	<0.001
Ventilator	0	33 (27.7)	<0.001
Death	0	23 (19.3)	<0.001

^*∗*^Respiratory distress syndrome, ^*∗∗*^standard deviation.

**Table 2:** Distribution of TNF-*α*-308 polymorphism, need for mechanical ventilator and surfactant and death in various categories.

Variable	Category	A/A	G/A	Total	*P* value
Surfactant	Yes	26 (92.9%)	2 (7.1%)	28 (100%)	0.271
	No	174 (82.9%)	36 (17.1%)	210 (100%)	

Ventilator	Yes	31 (93.9%)	2 (6.1%)	33 (100%)	0.094
	No	169 (82.4%)	36 (17.6%)	205 (100%)	

Death	Yes	22 (95.7%)	1 (4.3%)	23 (100%)	0.139
	No	178 (82.8%)	37 (17.2%)	215 (100%)	

**Table 3:** Distribution of IL-10-1082 polymorphism, need for mechanical ventilator and surfactant and death in various categories.

Variable	Category	G/G	G/A	Total	*P*
Surfactant	Yes	16 (57.1%)	12 (42.9%)	28 (100%)	0.319
	No	140 (66.7%)	70 (33.3%)	210 (10%)	

Ventilator	Yes	19 (57.6%)	14 (42.4%)	33 (100%)	0.299
	No	137 (66.8%)	68 (33.2%)	205 (100%)	

Death	Yes	13 (56.5%)	10 (43.5%)	23 (100%)	0.338
	No	143 (66.5%)	72 (33.5%)	215 (100%)	

**Table 4:** Frequency of genotype or allele of TNF-*α*-308 and IL-10-1082 polymorphism in different groups of pediatrics.

Polymorphism	Genotype/allele	Control (*N* (%))	RDS^*∗*^ (*N* (%))	*P*	OR (95% CI)
TNF-alpha-308	AA	98 (82.4)	102 (87.7)	0.479	0.778 (0.387–1.561)
	GA	21 (17.6)	17 (14.3)		
	A allele	217 (91.2)	221 (92.9)	0.499	0.795 (0.408–1.548)
	G allele	21 (8.8)	17 (7.1)		

IL-10-1082	GG	87 (73.1)	69 (58)	0.014	1.97 (1.143–3.397)
	GA	32 (26.9)	50 (42)		
	G allele	206 (86.6)	188 (79)	0.029	1.712 (1.053–2.783)
	A allele	32 (13.4)	50 (21)		

^*∗*^Respiratory distress syndrome.

**Table 5:** Result of logistic regression for dependent variable of groups.

Variable	Coefficient	SE^*∗*^ of coefficient	Sig.	OR	95% CI for OR	
					Lower	Upper
IL-10-1082	0.513	0.186	0.006	1.671	1.16	2.408
Gestational age	−1.303	0.189	<0.001	0.272	0.187	0.394
Sex	−0.699	0.352	0.047	0.497	0.25	0.991
Constant	45.663	6.679	<0.001	—	—	—

^*∗*^Standard error.
